# The Red Fox (*Vulpes vulpes*) as a Natural Reservoir of *Trichinella britovi* from North-Eastern Romania: Epidemiological and Molecular Evidence

**DOI:** 10.3390/vetsci13090964

**Published:** 2026-09-14

**Authors:** Olimpia Iacob, Laura Andreea Olariu, Gianluca Marucci, Francesco Celani, Ciprian Chiruță, Mihai Mareș

**Affiliations:** 1 Department of Clinics, Parasitology and Parasitic Diseases, Faculty of Veterinary Medicine, “Ion Ionescu de la Brad” Iași University of Life Sciences, 3 Aleea Mihail Sadoveanu, 700490 Iași, Romania; olimpia.iacob@iuls.ro; 2 Department of Infectious Diseases, European Union Reference Laboratory for Parasites, Instituto Superiore di Sanità, Viale Regina Elena 299, 00161 Rome, Italy; gianluca.marucci@iss.it (G.M.); francesco.celani@iss.it (F.C.); 3 Department of Exact Sciences, Faculty of Horticulture, “Ion Ionescu de la Brad” Iași University of Life Sciences, 3 Aleea Mihail Sadoveanu, 700490 Iași, Romania; ciprian.chiruta@iuls.ro; 4 Department of Public Health, Faculty of Veterinary Medicine, “Ion Ionescu de la Brad” Iași University of Life Sciences, 3 Aleea Mihail Sadoveanu, 700490 Iași, Romania; mihai.mares@iuls.ro

**Keywords:** red fox (*Vulpes vulpes*), epidemiology, sylvatic trichinellosis, multiplex PCR, *T. britovi*, North-Eastern Romania

## Abstract

Wild carnivores play an important role in maintaining *Trichinella* spp. infection in the sylvatic environment and may contribute to the transmission of the parasite to domestic animals and humans. Northeastern Romania hosts a large population of red foxes (*Vulpes vulpes*), a species not routinely included in trichinellosis surveillance programs. This study aimed to determine the geographical distribution and prevalence of *Trichinella* infection in red foxes and to identify the species involved. Between 2023 and 2025, muscle samples (tongue, diaphragm, and intercostal muscles) were collected from 617 red foxes culled as part of epidemiological surveillance activities. Samples were examined by direct trichinelloscopy, and positive specimens were further analyzed by multiplex PCR at the European Union Reference Laboratory for Parasites in Rome, Italy, for species identification. *Trichinella* infection was detected in foxes from all investigated counties of northeastern Romania, regardless of landforms. The overall prevalence was 24.3% (150/617). Of the 148 isolates subjected to multiplex PCR, 120 yielded positive amplification, all of which were identified as *Trichinella britovi*. The widespread distribution and high prevalence of *T. britovi* confirm the role of the red fox as an important natural reservoir, supporting the maintenance and circulation of the parasite in the sylvatic environment and highlighting the need for continued surveillance and further epidemiological studies.

## Simple Summary

Red foxes are widespread wild animals that can carry parasites capable of infecting other animals and, in some cases, humans. One of these parasites is *Trichinella*, which is transmitted to humans through the consumption of contaminated meat and causes trichinellosis. Although the parasite is mainly maintained in wildlife, infected wild animals may contribute to its spread to domestic animals. In northeastern Romania, where red foxes are abundant, their role in maintaining this parasite has not been routinely investigated. This study examined 617 red foxes collected between 2023 and 2025 to determine how common the parasite is and how widely it is distributed. About one in four foxes proved to be infected, and infected animals were found in all investigated areas, including mountainous, hilly, and lowland regions. All identified parasites belonged to the same species, *Trichinella britovi*. These findings show that red foxes are an important natural reservoir of this parasite and improve our understanding of its circulation in wildlife. This information is valuable for disease surveillance and for helping protect both animal and public health.

## 1. Introduction

Trichinellosis is a serious parasitic zoonosis with global spread, caused by the ingestion of raw or undercooked meat infected with larvae of the *Trichinella* genus, which parasitize autoheteroxenically in mammals and birds [1]. Taxonomically, the genus *Trichinella* is classified in the phylum Nematoda, class Enoplea, subclass Dorylaimia, order Trichinellida, family Trichinellidae [2] and includes, to date, 10 species and three genotypes. Depending on the presence of the collagen capsule around the larva in the muscle cell, the species are separated into two groups: encapsulated species and non-encapsulated species [3].

The encapsulated species are: *Trichinella spiralis* (T1), *Trichinella nativa* (T2), *Trichinella britovi* (T3), *Trichinella murrelli* (T5), *Trichinella nelsoni* (T7), *Trichinella patagoniensis* (T12), *T. chancalensis* (T13), and three genotypes (*Trichinella* T6, T8 and T9). The non-encapsulated species are: *Trichinella pseudospiralis* (T4), *Trichinella papuae* (T10), *Trichinella zimbabwensis* (T11); all *Trichinella* species infect humans [4]. In nature, the *Trichinella* genus has a larger natural reservoir in wildlife than in domestic animals, which complicates the epidemiological surveillance of trichinellosis [5]. In Europe, four species circulate more frequently: *T. spiralis*, *T. britovi*, *T. nativa* and *T. pseudospiralis* [6].

*T. britovi*, first reported by Pozio et al. (1992) [7], is an encapsulated, freeze-tolerant species that infects humans, with a wide geographical distribution: it starts in northwestern Africa, covers most of Europe and continues to Central Asia [8]. *T. britovi* parasitizes mainly in wild carnivores, being involved in the sylvatic cycle of trichinellosis throughout the European continent [9]. It is most commonly reported in wild boar and red fox, but also in other canids, felids, mustelids, ursids, beavers, rats and humans [10]. In red fox (*Vulpes vulpes*), *T. britovi* has been identified in most European countries: Austria, Bulgaria, Croatia, Czech Republic, Estonia, Finland, France, Germany, Greece, Hungary, Italy, Latvia, Lithuania, Netherlands, Norway, Poland, Portugal, Romania, Serbia, Slovakia and Switzerland [10]. The prevalence of *T. britovi* in red foxes in Europe varies across countries and geographical regions. Exceptionally high infection rates have been documented in Northern Europe: prevalence reached 96.4% in Latvia [11], and 69.0% in Estonia [12]. A high value was also observed in Sardinia, Italy, standing at 27.6% [13]. In Serbia, findings varied between 4.7% and 12.3% [14,15], with *T. britovi* accounting for 22.2% of the isolates. Similarly, Slovakia showed an average prevalence of 9.6%, with *T. britovi* representing 93.9% of the infections [16]. In Hungary, regional infection rates fluctuated between 1.8% and 6%, with *T. britovi* comprising 85.7% of the isolates [17]. In Poland, prevalence varied depending on the study, with reported values of 15.6% [18], 2.7% [19], and local ranges spanning from 0.5% to 7.7% [20]. Much lower prevalence levels were observed in Western and Southern mainland Europe, including 2.7% in France [21], 2.1% in northern Portugal [9], 1.1% in mainland Italy [22], and 0.31% in Germany [19]. Although *T. britovi* is frequently the dominant species, it can coexist with other species in foxes; specifically, co-infections or sympatric occurrences have been reported with *T. nativa* in Germany, Slovenia, and the Netherlands, and with *T. spiralis* in Germany and Poland [10].

In Romania, *T. britovi* is the most prevalent in wild animals [23], being identified in wolf (*Canis lupus*), golden jackal (*Canis aureus*) [24,25], wild cat (*Felis silvestris*), Eurasian lynx (*Lynx lynx*) [26], pine marten (*Martes martes*) [27], brown bear (*Ursus arctos*), wild boar (*Sus scrofa*) [28], European badger (*Meles meles*) [29], and red fox (*Vulpes vulpes*) [30,31].

The red fox (*Vulpes vulpes*), sentinel of the sylvatic cycle, is an opportunistic and scavenging predator, with a wide distribution throughout Romania, including rural and peri-urban areas. This aspect places it at the interference between the synanthropic cycle (predation on domestic animals or consumption of abandoned carcasses) and the sylvatic cycle (consumption of carcasses of wild boars, rodents or other wild carnivores) of *Trichinella* species. The red fox is a vital epidemiological indicator due to its population density and mobility, contributing to the conservation of *Trichinella* species, and their dissemination over large geographical areas [31]. In this context, northeastern Romania, which has varied landforms, ensures the existence of an abundant population of red foxes that has never been studied unitarily and is not included in the epidemiological surveillance activity of trichinellosis. The aim of this research is to determine, for the first time, the prevalence of *Trichinella* species in red foxes, in northeastern Romania, the geographical distribution of *Trichinella* infection, and the molecular identification of the *Trichinella* species involved.

## 2. Materials and Methods

### 2.1. Animals

A total of 617 red fox carcasses (578 adults and 39 young) obtained by random shooting during the winter months, as part of the epidemiological surveillance activity of rabies in northeastern Romania, were examined for *Trichinella* spp. infection by trichinelloscopy between 2023 and 2025.

### 2.2. Sampling Area

The study was conducted in northeastern Romania, within the historical region of Moldavia (Romanian: Moldova). The investigated area included eight counties—Suceava (SV), Botoșani (BT), Neamț (NT), Bacău (BC), Iași (IS), Vaslui (VS), Vrancea (VR), and Galați (GL)—covering a total area of 46,173 km^2^. Geographically, the region extends between 44.78° and 48.24° N latitude and 24.97° and 28.18° E longitude. The landscape is characterized by diverse geomorphological features, including the Carpathian Mountains (SV, NT, BC, VR counties) the Subcarpathian hills, plateaus, and lowland plains (BT, IS, VS, GL counties).

### 2.3. Sampling and Preservation of Samples

Muscle tissue samples (tongue, diaphragm, and intercostal muscles) were collected from each fox, with an average of 50 g of tissue obtained per animal. The samples were individually packaged in plastic bags, labeled, and stored at −80 °C, approximately 30 days, until laboratory examination.

### 2.4. Examination of Muscle Samples

Frozen samples were thawed gradually at 4 °C for 24 h before examination. Muscle tissues were examined for the presence of *Trichinella* larvae by direct trichinelloscopy.

Although, artificial digestion method is currently considered the gold standard (EU Regulation no. 1375/2015) according to the International Commission on Trichinellosis and European legislation, we used direct trichinelloscopy, since freezing muscle tissue samples at −80 °C inactivated the larvae in the *Trichinella* cysts, the risk of their digestion together with the muscle tissue during artificial digestion processes being real.

Muscle tissue fragments found positive were transferred to 1.5 mL microcentrifuge tube and refrozen at −80 °C until molecular analysis for species identification.

### 2.5. Molecular Identification of Trichinella Species

*Trichinella* positive muscle tissue fragments, refrozen at −80 °C, were submitted to the European Union Reference Laboratory for Parasites (EURL-P), Rome, Italy, for species identification by multiplex PCR [32]. Species identification of *Trichinella* larvae was done by multiplex polymerase chain reaction (Multiplex PCR) assay, at the European Union Reference Laboratory for Parasites (EURLP), Instituto Superiore di Sanita, Rome, Italy.

Because the samples had undergone repeated freezing and thawing cycles and consisted of very small muscle fragments, recovery of larvae by the standard HCl-pepsin artificial digestion method was not feasible. Therefore, DNA extraction was performed directly from encapsulated larvae present within the muscle tissue. Individual encysted larvae were carefully dissected from the surrounding muscle tissue using a sterile scalpel and transferred into clean 1.5 mL microcentrifuge tubes. For each positive fox, between three and five encysted larvae were collected and analyzed.

Genomic DNA was extracted using the DNA IQ System and Tissue and Hair Extraction kits (Promega, Madison, WI, USA). Species identification was performed by multiplex PCR using five primer pairs targeting the expansion segment V (ESV), ITS1, and ITS2 regions of the ribosomal DNA repeat, generating species-specific amplification profiles [33]. PCR products were analyzed by capillary electrophoresis using the QIAxcel Advanced System equipped with a DNA High Resolution Cartridge (Qiagen GmbH, Hilden, Germany). Electrophoretic separation was performed using the OM500 method with QIAxcel ScreenGel software (version 2.1), and fragment analysis was carried out using the QX Alignment Marker (15–600 bp) and the QX DNA Size Marker (50–800 bp).

### 2.6. Statistical Analysis

Confidence intervals (CI 95%) were calculated using the Wilson method for binomial proportions using Microsoft Excel 2016 Professional. Confidence Interval; α = 0.05 was considered as statistically significant.

To assess the differences in the prevalence of *Trichinella* spp. in red foxes, pairwise comparisons were performed between counties and between the years included in the study. For each comparison, the χ^2^ (Chi-square) test was applied to compare proportions, using the absolute frequencies of positive and negative foxes for *Trichinella* spp. infection, and not the percentage values of prevalence. The analysis was based on comparing the number of positive and, respectively, negative animals between the two compared categories. For each comparison, the *p*-value corresponding to the statistical test was calculated, and the statistical significance level was set at α = 0.05.

Given the large number of pairwise comparisons performed, the *p*-values obtained were adjusted using the Holm (Holm–Bonferroni) method implemented in MATLAB R2024a (MathWorks, Natick, MA, USA). This procedure involves ordering the *p*-values and sequentially applying adjusted significance thresholds. Differences between counties and between the analyzed years were considered statistically significant when the *p*-value adjusted by the Holm method was <0.05.

## 3. Results

### 3.1. Prevalence and Spatial Distribution of Trichinella spp. in Red Fox

**Figure 1:**
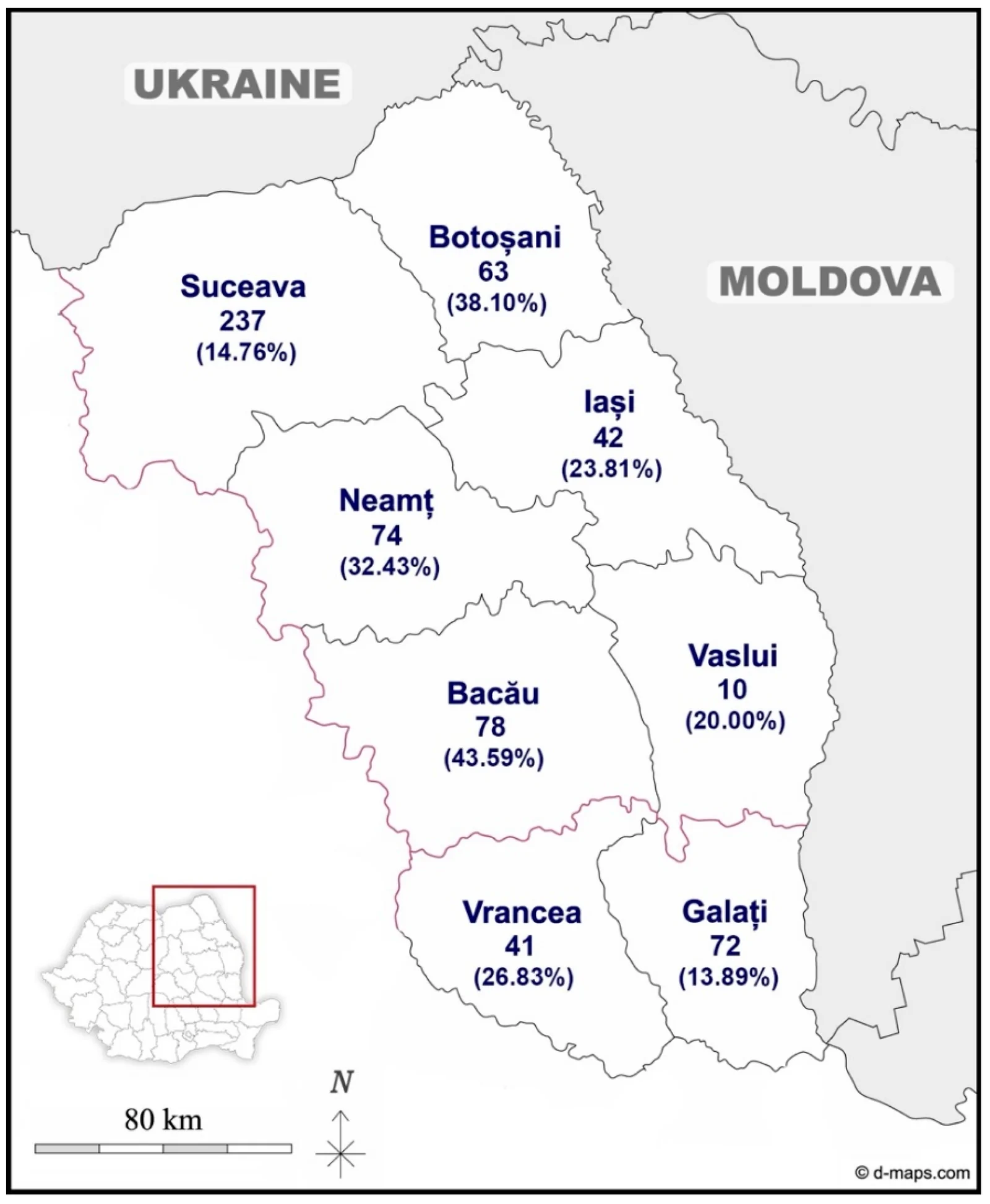
Spatial distribution of *Trichinella* infection in red foxes (*Vulpes vulpes*) from northeastern Romania during 2023–2025. For each county, the number of tested animals is reported, with the prevalence (%) given in brackets.

The overall prevalence of *Trichinella* infection in red foxes was 24.3% (150/617, CI 95%: 21.1–27.8%). The prevalence of *Trichinella* infection varied among the investigated areas, with positive red foxes identified in all eight counties (Table 1, Figure 1 and Figure S1). County-specific prevalence rates ranged from a minimum of 13.9%, CI 95%: 5.9–21.9 in GL to a maximum of 43.6%, CI 95%: 32.6–54.6 in BC. High endemicity was also evident in BT and NT, which exhibited prevalence rates of 38.1%, CI 95%: 26.1–50.1 and 32.4%, CI 95%: 21.8–43.1 respectively. The remaining counties, VR (26.8%: CI 95%: 13.3–40.4), IS (23.8%, CI 95%: 10.9–36.7), VS (20.0%, CI 95%: 4.8–44.8), and SV (14.8%, CI 95%: 10.3–19.3), showed intermediate levels of infection, confirming widespread circulation of the parasite throughout the entire northeastern region of Romania.

The prevalence of *T. britovi* in red foxes in mountainous counties compared to hilly and plain counties, as well as the statistical significance, are presented in Table 2.

The annual and overall prevalence of *Trichinella* infection during the study period (2023–2025) is presented in Table 3 and Figure S2. A distinct decreasing trend was observed, with the highest annual prevalence recorded in 2023 at 52.7%, CI 95%: 41.3–63.9. In the following years, the infection rate decreased progressively, dropping to 23.9%, CI 95%: 19.2–29.0 in 2024, and reaching its lowest value in 2025 at 16.3%, CI 95%: 12.0–21.3.

### 3.2. Molecular Identification of Trichinella spp. in Red Fox

**Figure 2:**
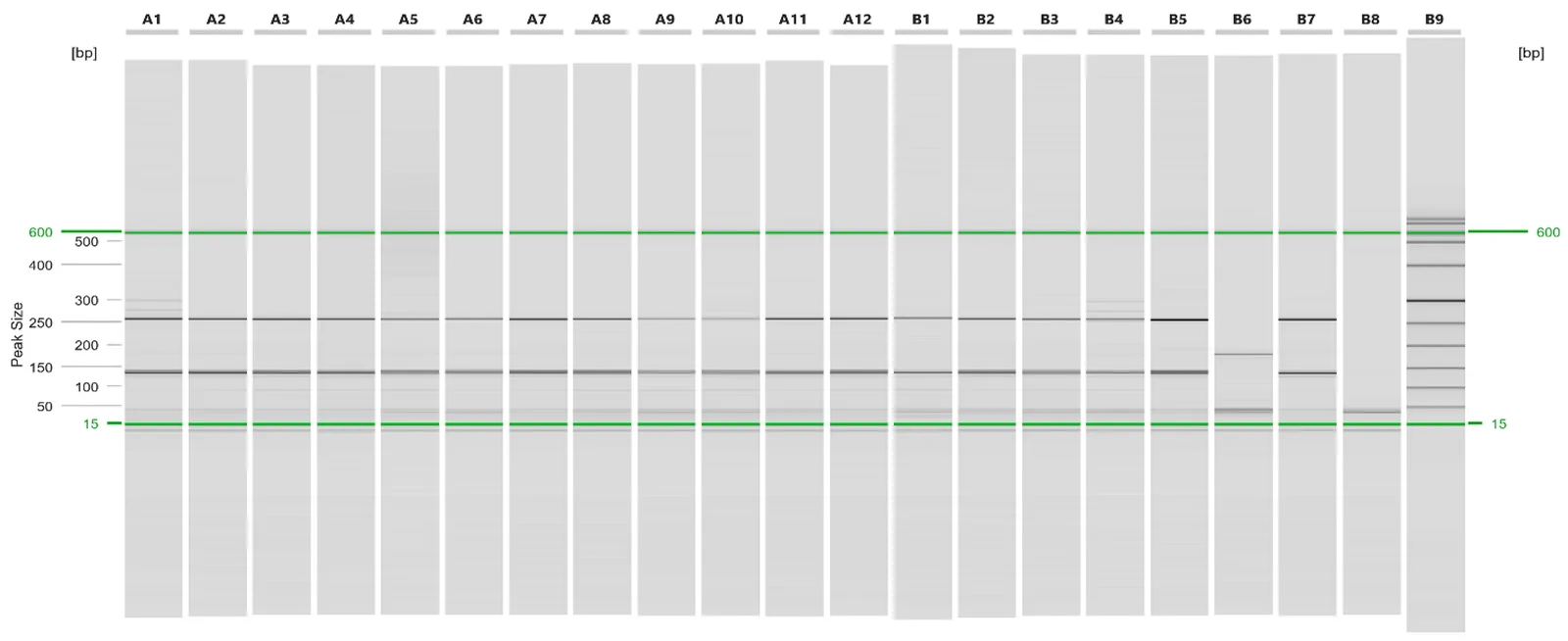
Representative capillary electrophoresis of multiplex PCR products for *Trichinella* species identification. Lanes A1 to B5: 2025 Romanian red fox isolates from Botoșani, Iași, Bacău, and Vaslui counties; Lane B6: *T. spiralis* positive control (reference larva) for the DNA purification step; Lane B7: *T. britovi* positive control (reference DNA) for the PCR; Lane B8: PCR negative control; Lane B9: size marker.

Out of the 148 *Trichinella* isolates tested by multiplex PCR, 120 (81.08%) yielded a positive result, all of which were identified as *T. britovi*. It is possible that the 28 positive samples that did not yield a PCR result contained other species of Trichinella while two other positive samples were unsuitable for multiplex PCR. In particular, amplification success rates varied across the study years: positive results were obtained for 21 out of 39 isolates (53.85%) in 2023 (Figures S3 and S4), 58 out of 67 isolates (86.57%) in 2024 (Figures S5 and S6), and 41 out of 42 isolates (97.62%) in 2025 (Figures S7 and S8). A representative capillary electrophoresis showing the specific amplification bands for *T. britovi* is displayed in Figure 2 and Figures S3–S8.

## 4. Discussion

Wild carnivores play an essential role in maintaining the sylvatic cycle of *Trichinella* spp., contributing to parasite persistence in natural ecosystems and providing a continuous source of infection for wildlife, domestic animals, and occasionally humans [34].

Romania’s extensive and heterogeneous ecosystems support abundant populations of wild carnivores, including wolves, lynx, golden jackals, and red foxes, which contribute to the maintenance and circulation of *Trichinella* spp. [35].

The distribution of *Trichinella britovi* in Romania is influenced by habitat characteristics, altitude and landforms. The parasite is predominantly associated with mountainous and hilly ecosystems [5,28,36]. Mountainous regions represent an important ecological reservoir, with *T. britovi* previously detected exclusively in mountainous areas and in wildlife hosts such as wild boar (*Sus scrofa*) and brown bear (*Ursus arctos*) [28]. The high abundance of large carnivores and omnivores involved in the sylvatic cycle, together with extensive forest cover and lower temperatures that favor larval survival in carcasses and carrion, may contribute to this pattern [8,26,37].

Hilly regions constitute a transitional zone where *T. britovi* may overlap with *T. spiralis*, reflecting interactions between sylvatic and synanthropic transmission cycles. Its occurrence at intermediate altitudes is also supported by its detection in hosts, such as the pine marten (*Martes martes*), which is widely distributed in Romanian forests [27].

Although *T. britovi* appears less frequent in lowland ecosystems, its circulation has also been documented in the raccoon dog in the Danube Delta and Danube and Prut floodplains [38]. Widely distributed carnivores such as the golden jackal (*Canis aureus*) and the red fox, may further contribute to maintaining transmission in these areas [15,39]. The red fox is particularly valuable as a sentinel host because of its wide distribution, high population density, opportunistic feeding behavior, and scavenging habits, which facilitate connections between sylvatic and domestic transmission cycles [31,40].

Previous studies have demonstrated substantial *Trichinella* infection in Romanian red foxes. *T. britovi* was identified in 24 of 25 foxes examined in western Romania [40], while prevalence values of 16% and 15.8% were reported in Transylvania and central Romania, respectively [23,30].

In western Romania, *T. britovi* prevalence reached 28.12% [31] The present study, complements these findings by demonstrating widespread *T. britovi* infection in red foxes from northeastern Romania. Overall, 150 of 617 examined foxes were positive, corresponding to a prevalence of 24.3%. Positive animals were detected in all investigated counties and across different landforms, indicating extensive circulation of *T. britovi* in northeastern Romania. County-level prevalence varied considerably ranging from 14.8% to 43.6% in Bacău among predominantly mountainous counties, and from 13.9% in Galați to 38.1% in Botoșani among areas characterized by hills, plateaus plains, and lowlands. Significant differences were observed between Suceava and Botoșani (*p* < 0.001), Suceava and Neamț (*p* = 0.018), Suceava and Bacău (*p* < 0.001), Botoșani and Galați (*p* = 0.029), and Bacău and Galați (*p* = 0.002). However, prevalence did not differ significantly between predominantly mountainous counties (24.2%) and hilly/plain counties (24.6%; *p* = 0.925). These findings indicate that, despite the recognized association of *T. britovi* with mountainous habitats, the parasite is broadly distributed across northeastern Romania.

The prevalence recorded in northeastern Romania is comparable with values reported elsewhere in the country [23,30,31,40]. A similar geographical pattern has been described in Slovakia, where prevalence was lower in lowland areas (6.9%) and higher in mountainous regions (14.2–25.2%), with an overall prevalence of 15.6% [41]. In a larger Slovak survey, prevalence increased from 4.9% in 2000 to 20.5% in 2007, confirming the widespread circulation of *Trichinella* in red foxes [42].

Marked annual variation was observed in the present study, with prevalence decreasing from 52.7% (39/74) in 2023 to 23.9% (71/297) in 2024, and 16.26% (40/246) in 2025. Differences between all three years were statistically significant: 2023 vs. 2024 (χ^2^ = 25.87; pHolm < 0.001), 2023 vs. 2025 (χ^2^ = 35.58; pHolm < 0.001), and 2024 vs. 2025 (χ^2^ = 4.83; pHolm = 0.028). This progressive decline should be interpreted cautiously because annual differences may reflect variation in sampling intensity, county representation, fox population density and distribution, ecological conditions, hunting pressure, prey availability, and stochastic fluctuations.

Nevertheless, infected foxes were detected throughout the study period, confirming the persistent presence of *T. britovi* in northeastern Romania.

Molecular investigations of selected positive samples consistently identified only *T. britovi,* with no evidence of mixed infections (Figure 2 and Figures S3–S8). The absence of *T. spiralis* or *T. pseudospiralis* was unexpected, particularly because mixed infections involving *T. spiralis* have been reported in Poland and *T. nativa* in Germany and Slovenia.

Other *Trichinella* species cannot, however, be completely excluded because molecular amplification failed in 28 trichinelloscopy-positive samples and two additional positive samples became unsuitable for molecular analysis. The unsuccessful PCR results may have resulted from DNA degradation following repeated freezing and thawing or PCR inhibition. The samples originated mainly from Botoșani and Bacău in 2023, Vrancea and Suceava in 2024, and Bacău in 2025, and were subject to multiple handling and temperature changes before molecular examination, potentially affecting DNA quality.

Although *T. britovi* prevalence of 36.76% has previously been reported in brown bears from the same geographical area [28], the red fox represents an important sentinel host for surveillance of the sylvatic cycle because of its abundance, ecological adaptability, mobility, and scavenging behavior. The present findings demonstrate widespread and persistent circulation of *T. britovi* in red foxes in northeastern Romania, highlighting their epidemiological importance in maintaining transmission among wildlife and their potential role in bridging sylvatic and domestic transmission cycles.

## 5. Conclusions

*T. britovi* was the only species identified in red foxes sampled in the northeastern part of Romania so far. Its high prevalence highlights the significant circulation of the species within the sylvatic environment and confirms the epidemiological relevance of the red fox as a sentinel host for monitoring *Trichinella* infection. The geographical distribution of positive cases across all investigated counties indicates a widespread and persistent circulation of *T. britovi* in red fox population. These findings suggest the need for continued epidemiological surveillance of the red fox population, as an important indicator, to better assess the dynamics of *T. britovi* circulation in wildlife and the potential risk of transmission to other animals.

## Figures and Tables

**Table 1:** Prevalence of *Trichinella* spp. in red foxes in northeastern Romanian counties during 2023–2025.

County	Tested Foxes	Positive Foxes	Prevalence (%)	CI 95%
Suceava (SV)	237	35	14.8	10.3–19.3
Botoșani (BT)	63	24	38.1	26.1–50.1
Neamț (NT)	74	24	32.4	21.8–43.1
Iași (IS)	42	10	23.8	10.9–36.7
Bacău (BC)	78	34	43.6	32.6–54.6
Vaslui (VS)	10	2	20.0	4.8–44.8
Vrancea (VR)	41	11	26.8	13.3–40.4
Galați (GL)	72	10	13.9	5.9–21.9
Total prevalence	617	150	24.3	21.1–27.8

C.I. 95% (Confidence Interval; α = 0.05 was considered as statistically significant).

**Table 2:** Prevalence of *Trichinella* spp. in red foxes in mountainous counties versus hilly and plain counties in northeastern Romania during 2023–2025.

County	Tested Foxes	Positive Foxes	Prevalence (%)	CI 95%	*p*-Value
Mountainous counties					
Suceava (SV)	237	35	14.8	10.3–19.3	0.925 (>0.05)
Neamț (NT)	74	24	32.4	21.8–43.1	
Bacău (BT)	78	34	43.6	32.6–54.6	
Vrancea (VR)	41	11	26.8	13.3–40.4	
Total	430	104	24.2	20.4–28.5	
Hilly and plain counties					
Botoșani (BT)	63	24	38.1	26.1–50.1	0.925 (>0.05)
Iași (IS)	42	10	23.8	10.9–36.7	
Vaslui (VS)	10	2	20.0	4.8–44.8	
Galați (GL)	72	10	13.9	5.9–21.9	
Total	187	46	24.6	19.0–31.3	

Statistical significance level: α = 0.05.

**Table 3:** Annual and overall prevalence of *Trichinella* spp. in red foxes in northeastern Romania during 2023–2025.

Category/Year	2023	2024	2025	Total
Tested foxes	74	297	246	617
Positive foxes	39	71	40	150
Prevalence (%)	52.7	23.9	16.3	24.3
Confidence interval (CI) 95%	41.3–63.9	19.2–29.0	12.0–21.3	21.1–27.8

## Data Availability

The original contributions presented in this study are included in the article/Supplementary Material. Further inquiries can be directed to the corresponding author.

## Supplementary Materials

The following supporting information can be downloaded at: https://www.mdpi.com/article/10.3390/vetsci13090964/s1, Figure S1: Spatial distribution of *Trichinella* spp. in red foxes, in northeastern Romania during 2023–2025; Figure S2: Annual and overall prevalence of *Trichinella* spp. in red foxes, in northeastern Romania during 2023–2025; Figure S3: Capillary electrophoresis profile of multiplex PCR products amplified from *Trichinella* larvae isolated from red foxes in 2023. A1–A10: isolates from Botoșani; A11–A12: isolates from Bacău; B1: *T. spiralis* reference DNA (positive control for the DNA purification step); B2: *T. britovi* reference DNA (PCR positive control); B4: PCR negative control; B9: DNA size marker. Figure S4: Capillary electrophoresis profile of multiplex PCR products amplified from *Trichinella* larvae isolated from red foxes in 2023. A1–C3: isolates from Bacău; C4: *T. spiralis* reference DNA (positive control for the DNA purification step); C5: *T. britovi* reference DNA (PCR positive control); C6: PCR negative control; C7: DNA size marker. Figure S5: Capillary electrophoresis profile of multiplex PCR products amplified from *Trichinella* larvae isolated from red foxes in 2024. A1–A10: isolates from Botoșani; A11–B3: isolates from Iași; B4–C1: isolates from Vrancea; C2 *T. spiralis* reference DNA (positive control for the DNA purification step); C3: *T. britovi* reference DNA (PCR positive control); C4: PCR negative control; C5: DNA size marker. Figure S6: Capillary electrophoresis profile of multiplex PCR products amplified from *Trichinella* larvae isolated from red foxes in 2024. A1–B3: isolates from Suceava; B4–D1: isolates from Neamț; D2 *T. spiralis* reference DNA (positive control for the DNA purification step); D3: *T. britovi* reference DNA (PCR positive control); D4: PCR negative control; D5: DNA size marker. Figure S7: Capillary electrophoresis profile of multiplex PCR products amplified from *Trichinella* larvae isolated from red foxes in 2025. A1–A10: isolates from Galați; A11–A12: isolates from Suceava; B1 *T. spiralis* reference DNA (positive control for the DNA purification step); B2: *T. britovi* reference DNA (PCR positive control); B3: PCR negative control; B4: DNA size marker. Figure S8: Capillary electrophoresis profile of multiplex PCR products amplified from *Trichinella* larvae isolated from red foxes in 2025. A1–A10: isolates from Suceava; B1: *T. britovi* reference DNA (PCR positive control); B2: PCR negative control; B3: DNA size marker.

## Abbreviations

The following abbreviations are used in this manuscript:

PCR	Polymerase Chain Reaction
*T.*	*Trichinella*
CI	Confidence Interval
