# Supplementary material for: The Red Fox (Vulpes vulpes) as a Natural Reservoir of Trichinella britovi from North-Eastern Romania: Epidemiological and Molecular Evidence
Source: Vet Sci. 2026 Sep 14;13(9):964. doi: 10.3390/vetsci13090964 (PMC13611997; doi:10.3390/vetsci13090964)
Supplement: Supplementary file 1 [file vetsci-13-00964-s001.zip › vetsci-4490258-supplementary.pdf]

Supplementary Materials: Figures S1–S8

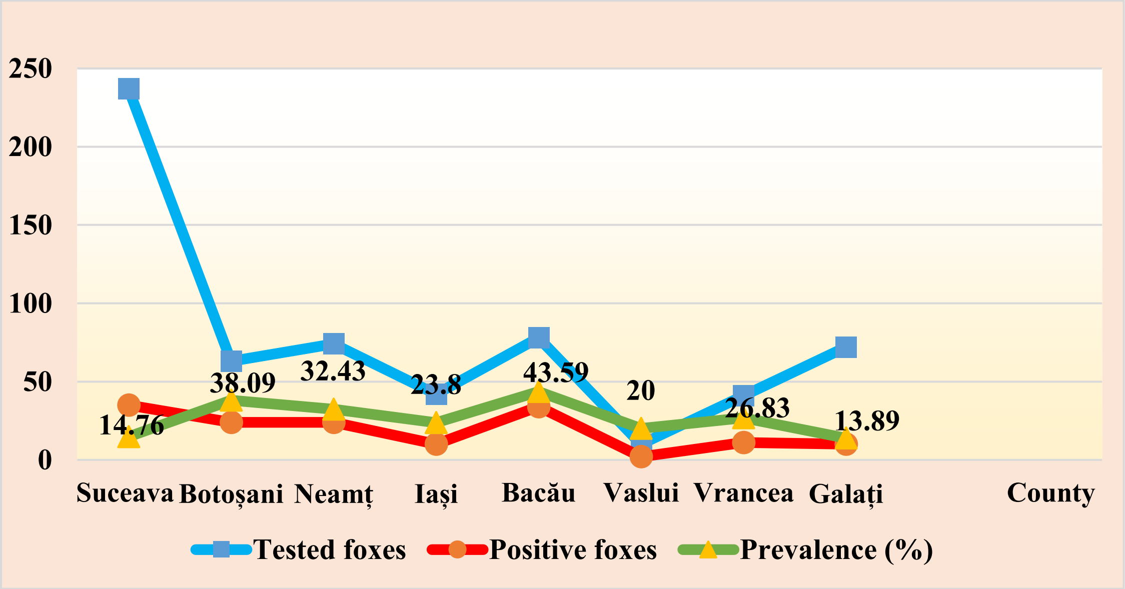

Figure S1. **Spatial distribution of *Trichinella* spp. in red foxes, in northeastern Romania during 2023–2025.**

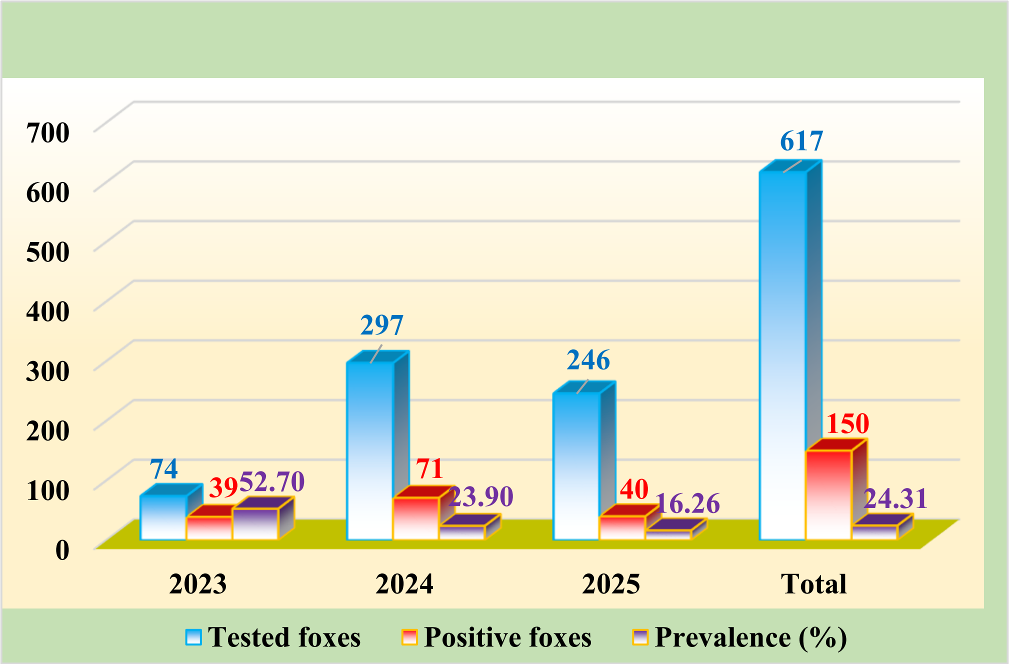

Figure S2. **Annual and overall prevalence of *Trichinella* spp. in red foxes, in northeastern Romania during 2023–2025.**

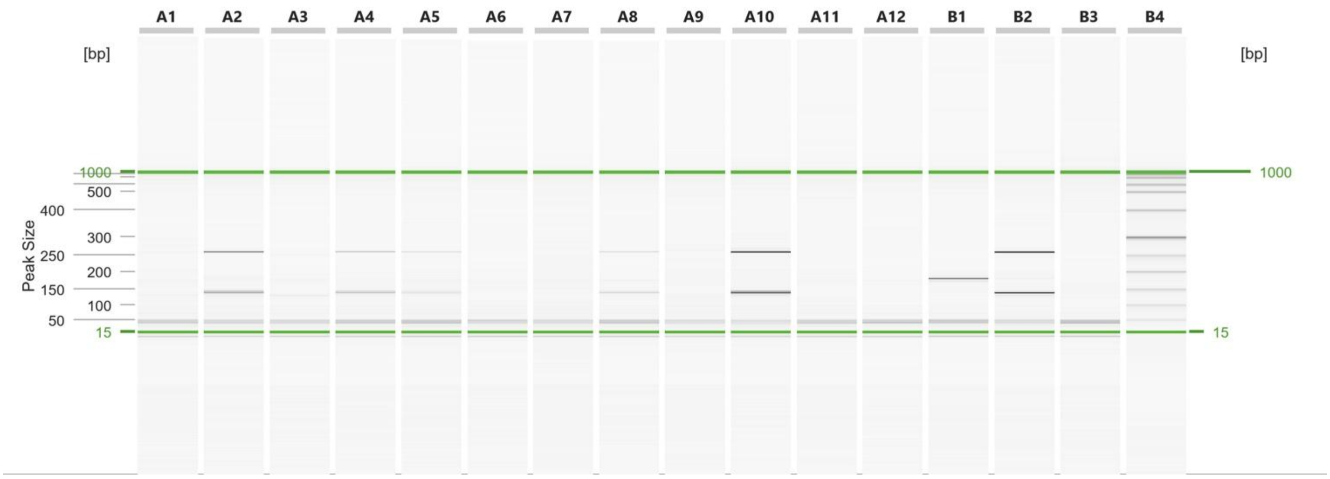

Figure S3. **Capillary electrophoresis profile of multiplex PCR products amplified from *Trichinella* larvae isolated from red foxes in 2023. A1–A10: isolates from Botoșani; A11–A12: isolates from Bacău; B1: *T. spiralis* reference DNA (positive control for the DNA purification step); B2: *T. britovi* reference DNA (PCR positive control); B4: PCR negative control; B9: DNA size marker.**

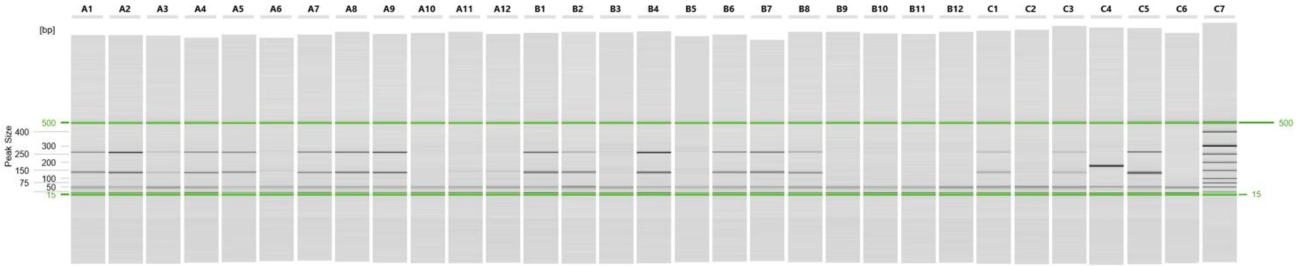

Figure S4. **Capillary electrophoresis profile of multiplex PCR products amplified from *Trichinella* larvae isolated from red foxes in 2023. A1–C3: isolates from Bacău; C4: *T. spiralis* reference DNA (positive control for the DNA purification step); C5: *T. britovi* reference DNA (PCR positive control); C6: PCR negative control; C7: DNA size marker.**

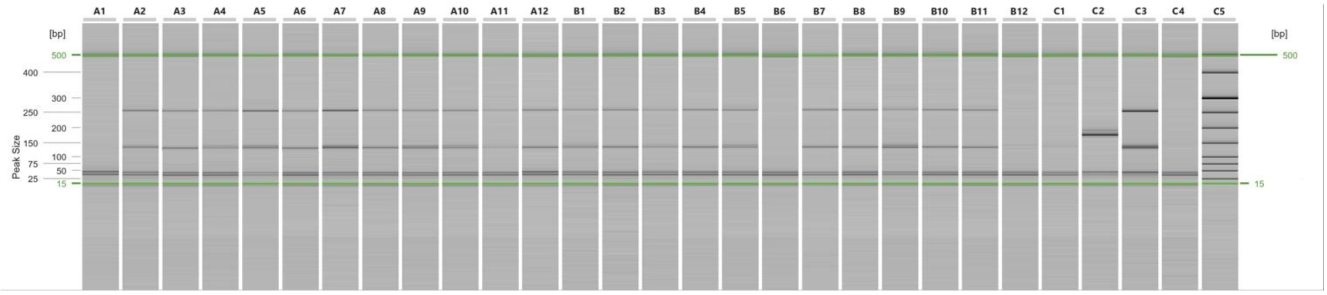

Figure S5. **Capillary electrophoresis profile of multiplex PCR products amplified from *Trichinella* larvae isolated from red foxes in 2024. A1–A10: isolates from Botoșani; A11-B3: isolates from Iași; B4-C1: isolates from Vrancea; C2 *T. spiralis* reference DNA (positive control for the DNA purification step); C3: *T. britovi* reference DNA (PCR positive control); C4: PCR negative control; C5: DNA size marker.**

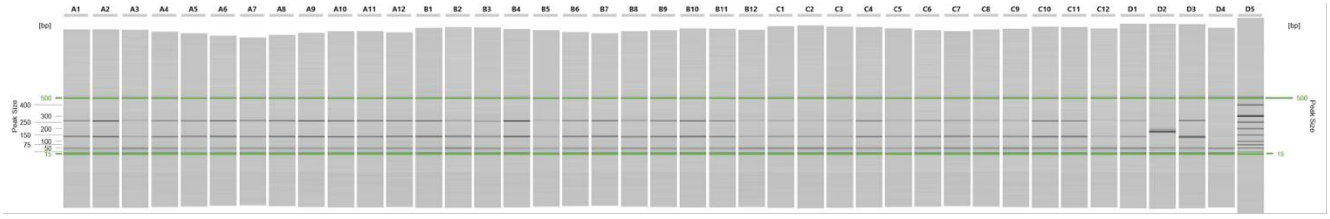

Figure S6. Capillary electrophoresis profile of multiplex PCR products amplified from *Trichinella* larvae isolated from red foxes in 2024. A1–B3: isolates from Suceava; B4-D1: isolates from Neamț; D2 ***T. spiralis*** **reference DNA (positive control for the DNA purification step); D3: *T. britovi* reference DNA (PCR positive control); D4: PCR negative control; D5: DNA size marker.**

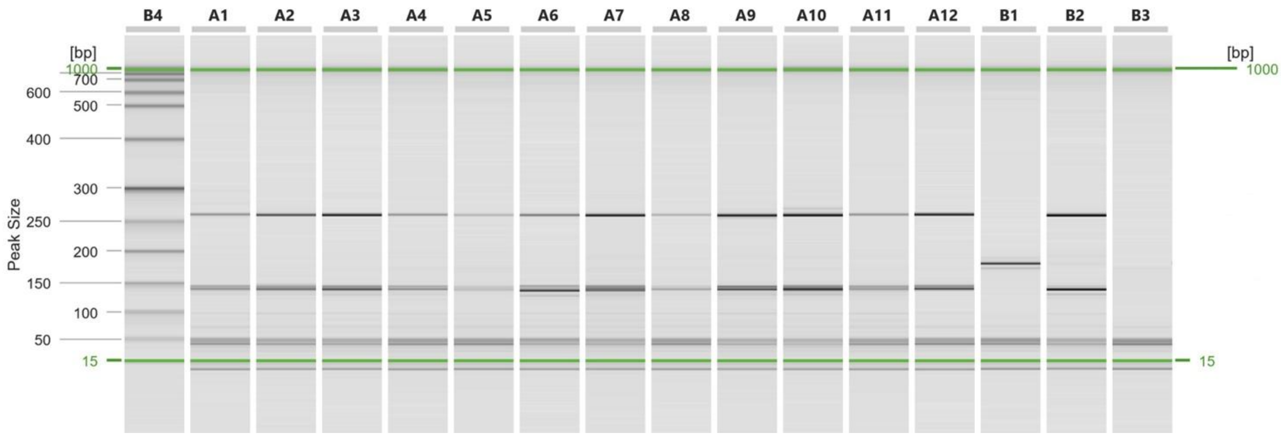

Figure S7. Capillary electrophoresis profile of multiplex PCR products amplified from *Trichinella* larvae isolated from red foxes in 2025. A1–A10: isolates from Galați; A11-A12: isolates from Suceava; B1 *T. spiralis* reference DNA (positive control for the DNA purification step); B2: *T. britovi* reference DNA (PCR positive control); B3: PCR negative control; B4: DNA size marker.

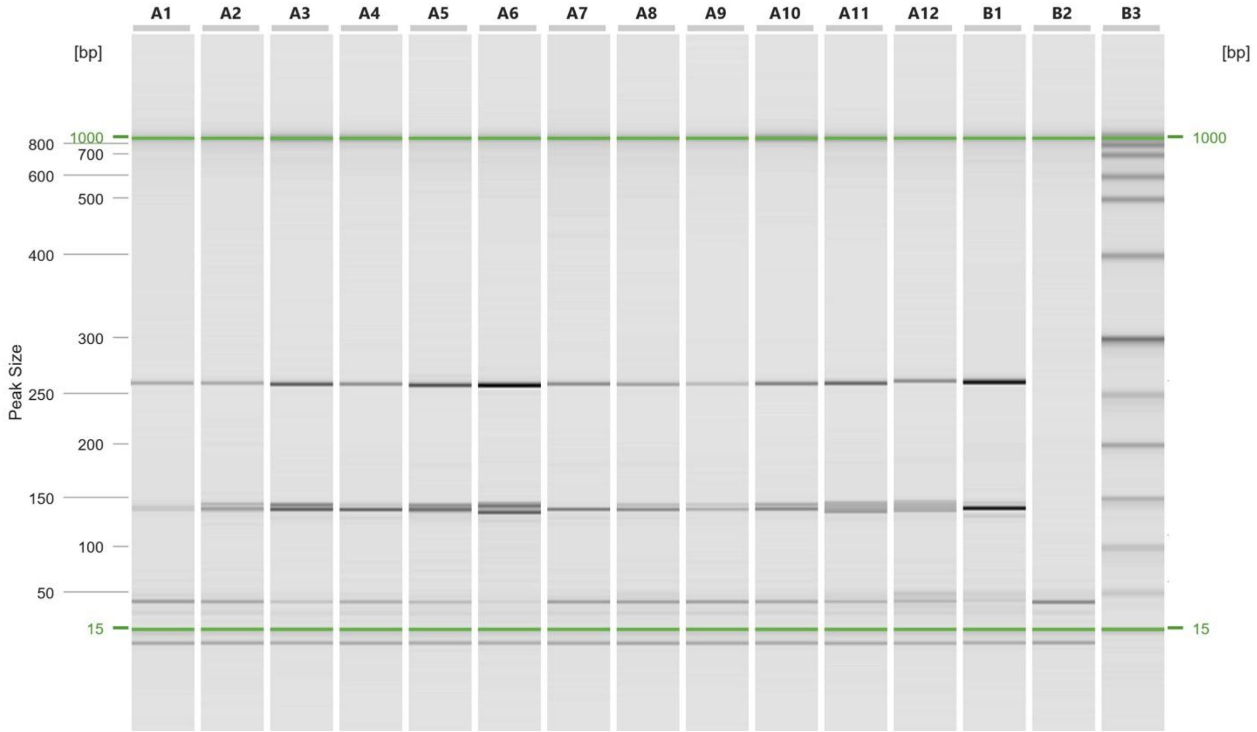

Figure S8. Capillary electrophoresis profile of multiplex PCR products amplified from *Trichinella* larvae isolated from red foxes in 2025. A1–A10: isolates from Suceava; B1: *T. britovi* reference DNA (PCR positive control); B2: PCR negative control; B3: DNA size marker.
